# Effect of Tualang honey on the anastomotic wound healing in large bowel anastomosis in rats-A randomized controlled trial

**DOI:** 10.1186/s12906-016-1003-6

**Published:** 2016-01-23

**Authors:** Muhammad Izani Aznan, Omaid Hayat Khan, Allah Obhayo Unar, Sharifah Emilia Tuan Sharif, Amer Hayat Khan, Syed Hassan Syed Abd. Aziz, Andee Dzulkarnaen Zakaria

**Affiliations:** 1 Department of Surgery, School of Medical Sciences, Universiti Sains Malaysia, Health Campus, Kota Bharu, 16150 Malaysia; 2 Discipline of Clinical Pharmacy, School of Pharmaceutical Sciences, Universiti Sains Malaysia, Penang, 11800 Malaysia; 3 Department of Histopathology, School of Medical Sciences, Universiti Sains Malaysia, Health Campus, Kota Bharu, 16150 Malaysia

**Keywords:** Anastomosis, Anastomotic bursting pressures, Honey, Wound healing

## Abstract

**Background:**

Honey has long been used for the treatment of number of ailments and diseases including surgical wounds. Current study evaluates the effectiveness of Tualang honey (TH) for large bowel anastomotic healing in Wistar rats.

**Methods:**

Thirty male Wistar rats were given a 3 centimeter infra-umbilical laparotomy wound, in`flicted on their abdomen. The colonic transection was performed at 5 cm distal to caecum, with end to end anastomosis of colon segment. They were divided into two groups. Group I was fed with standard rat chow and water. Meanwhile, Group II apart from standard feed, was also given TH 1.0 g/kg every morning until day seven post operatively. Afterwards, anastomotic bursting pressures were measured and histopathological examination on the anastomosis line was performed with light microscopes. The data from two groups were analyzed by Independent paired t test for continuous variables.

**Results:**

It was found that the tensile strength of colon anastomosis (95 % CI; *p* = <0.001) and the histopathological study including fibroblast count (*p* = <0.001) and inflammatory cells (*p* = 0.002) showed statistically significant difference in the favor of TH-treated group. Meanwhile, neovascularization formation was not statistically significant (*p* = 0.807); however, the overall count in the TH group was high.

**Conclusion:**

Oral treatment with TH enhances anastomotic wound healing by increasing the number of fibroblasts and by decreasing inflammatory cells leading towards increased wound strength.

## Background

Since the very beginning of 17th century, intestinal anastomosis procedure is being practiced and has been considered a promising surgical intervention for patients with diseased bowels. However, a disastrous complication of intestinal anastomosis is anastomotic leak resulting in peritonitis, which is associated with high mortality, morbidity and increased hospital stay [1]. Apart from modern surgical techniques and precautions, intra-abdominal adhesion formation after surgery is still an obvious manifestation [2]. Adhesion formation has similar pathways as that of wound healing thus modulation of the release factors could be expected to affect its formation [3, 4]. The incidence of anastomotic leak varies widely with a usual range between 2.7 % to 26 % in abdominal surgeries [1, 5], risk factors for which normally remain type of procedure, patient demographics and surgical techniques. [6, 7] A study by Neil Hyman et al. showed that leakage of intestinal anastomosis was diagnosed clinically ranging from day one to nineteenth day post operatively [5]. Mortality rates associated with anastomotic leak after surgery range from 6.2 % to 37 % [8, 9]. The onset of anastomotic leakage increases the risk of mortality four to seven-folds [10, 11].

Various methods including perioperative management, careful operative techniques, post-operative care and use of different healing agents such as honey, Aloe Vera, snakehead fish (Haruan) are being practiced in order to decrease the incidence of the leak and/or to facilitate the anastomotic healing process, thus improving patient outcome [12–15]. Honey has a long history of traditional use for wound healing and has been referred extensively in the medical literature since ancient times. Emre A et al. in 2010 showed that honey improved anastomotic wound healing in rats [16]. In many different animal studies, honey indeed has been proven effective, as in studies by Subrahmanyam in 1996 showed that wound dressing with honey healed faster [17].

The aim of the present study is to investigate the effect of TH in large bowel anastomosis in rats, undertaking its effect on tensile strength of large bowel anastomosis by measuring the bursting pressure and its effect on histopathology examination.

## Methods

A Randomized Control Trial study was conducted over a period of eight months at the Animal Research and Service Centre (ARASC) of Universiti Sains Malaysia, Kubang Kerian. Tualang honey was supplied by the Federal Agriculture Marketing Authority (FAMA) office in Kedah, Malaysia. Study was approved by the Animal Ethical Committee and the sample size of 30 rats was obtained using certified software (PS Software). By using block randomization, all rats (*N* = 30) were categorized into two groups (t = 2); one control group and the other as study group which was administered Tualang honey (TH) orally. So rats were divided into 15 nomogenous groups with 2 rats in each group. One of the rats in individual nomogenous group was randomly assigned as a control and the other one as the study. Colonic resection and end to end bowel anastomosis was performed on all the thirty rats.

### Materials

#### Tualang Honey

In Malaysia, there are varieties of honey available depending on the type of bees and where they make their combs. The most famous of which is TH, which gets its name from the rock bees (*Apis dorsata*) [18] that make their hives high up on the branch of Tualang tree (*Kompassia excelsa*) located in the north west region of Peninsular Malaysia [19]. It has been used traditionally in the local community for the various purposes including treatment for many diseases, as a food, supplement and also as a preservative. The TH is expensive compared to other honey as it is the best among local honey due to its outstanding therapeutic, anti-inflammatory, anti-oxidant, immunity enhancing, anti-tumor, anti-diabetic and anti-bacterial qualities including its activity against number of gram positive as well as gram negative species including *Klebsiella pneumonia,Pseudomonas spp.and Acinetobacter baumannii* [20]. Apart from its effect on wound management, TH also provides an additional antioxidant effect to many organs for example pancreas especially in diabetic rat model. This protective effect on pancreas against oxidative stress might also partially contribute to the hypoglycemic effect of TH in diabetic model [14].

#### Animals

Thirty male Wistar rats at the age of 90 days, weighing about 250 - 400gm were classified for the study. All thirty male rats were kept in ARASC one week prior to study and were fed with free access to standard commercial diet and water ad libitum throughout the study and were kept in separate cages under constant temperature of 21 to 22^○^C to maintain healthy and clean environment. No mechanical bowel preparation was done prior to the experimental procedure and neither any intra-operative bowel irrigation was performed.

##### Surgical Procedure:

The procedure was conducted in several sessions for all the thirty rats in both groups. The rats were anesthetized with an intramuscular Ketamine (Troy Lab. Pty Ltd, Australia) 60 mg/kg of the body weight. This anesthesia was given by a specialized team from the department of veterinary medicine of Universiti Sains Malaysia. The abdomen was shaved during anesthesia and the rats were placed and secured in dorsum recumbence position. When fully anaesthetized, the shaved area was cleaned and the operation site was isolated with sterile towel. Afterwards the surgical procedure was performed under aseptic conditions.

A 3 cm incision initiating from the infra umbilicus, at the midline, along the line of linea alba was made. Abdomen was opened in layers until the peritoneum was exposed and then the caecum was identified. Large bowel was transected at 5 cm distal to caecum. End to end anastomosis of transected colon was performed using 6/0 Premilene® (B Braun Aesculap, Germany) in single layer interrupted fashion. This suture was selected as it is commonly used and is monofilament (less tissue damage). The abdominal muscle layer was closed as continuous sutures with 4/0 Premilene® (B Braun Aesculap, Germany) and the same type of sutures were used to close the skin and subcutaneous tissue layer. Post-operative dressing, in the form of abdominal bandage, was applied to prevent self-mutilation. Right after the procedure, they were monitored for spontaneous breathing effort and movement and their daily activity and feeding habits were also observed.

After the surgical procedure was completed on all the thirty rats, they were kept in an individual cage according to their group and number. Fifteen rats in control group (Group I) were fed with standard rat chow and water, while other fifteen rats in the study group (Group II) were given Tualang honey (TH) apart from standard rat chow and water. Tualang honey (1.0 g/kg) was given orally (once daily, every morning) until the seventh day post-operatively. On the seventh day after the procedure, all the rats were sacrificed. The rats were euthanized using carbon monoxide gas until death. An incision was made on the same anterior abdominal wall wound and the anastomotic site was exposed outside the abdominal cavity. Large bowel was resected 2.5 cm proximally and distal to the anastomotic site from all the animals and were tested for biomechanics and histology accordingly.

##### Tensile strength measurement:

In both groups, 5 cm segment was excised proximally and distal to the anastomotic site, positioning it in the center. Feces that were contained inside the colon segment were gently removed. The suture at the anastomotic site was not removed. One end of the excised segment was tied and the other end was catheterized with polyurethane tube (Fore Sight Industries, Sdn Bhd, Malaysia) and stay-suture was tied circumferentially incorporating both tissue and the tube so as to prevent air leakage. The external end of the tube, a syringe (Terumo Corp. Tokyo Japan) with infusion pump (Teruma Corp. Tokyo, Japan) and a mercury sphygmomanometer (Accoson Ltd, United Kingdom) were all connected to each other by 3 way of T-shaped adapter. The colon segment was then placed in a container filled with saline and room air was pumped into tube at a rate 5 ml/min using infusion pump. The manometer reading at the instant of sudden pressure decrease or when bubbles were seen was recorded as the bursting pressure. The maximum pressure reading before the pressure declined suddenly was recorded as the bursting pressure [21]. The suture at the anastomotic site was not removed during this process. The equipment used for bursting pressure measurement was setup using local material because anastomotic sample was small in size.

##### Histopathology examination:

The tissue specimens (5 cm colonic segment with the anastomosis site in the center in 10 % formaline) were sent to the histopathological laboratory on the same day of procedure. The specimens were processed for slide examination. The slides obtained were examined under the light microscope (Olympus CX 31) over a high power field (40×) by a single pathologist who was assigned to help in specimen analysis. Examination was done over ten fields per specimen in a random fashion and an average was taken as the final count. Hematoxylin and Eosin stains (H&E stain) were used and the cells were seen in all layers of the colon and they included fibroblast count, inflammatory cells (abundant lymphoplasma cells, macrophages and polymorphonuclear leucocytes) and blood vessels which were counted semi quantitatively and graded histologically in a blind fashion, using the 0 – 4 Ehrlich and Hunt numerical scale (Table 1) [22].

**Table 1:** Histological grading scale [22]

Grade	Histologic analysis
0	No evidence
1+	Occasional evidence
2+	Light scattering
3+	Abundant evidence
4+	Confluence cells or fibres

##### Statistical Analysis:

All data were expressed as mean ± Standard Deviation (SD) and tabulated in the form of table (Table 2). The Statistical Package for the Social Science (SPSS) version 18.0 for windows was used in statistical analysis. The data from two groups were analyzed by Independent paired t test for continuous variables. Difference was considered statistically significant when *P* <0.05.

**Table 2:** Results of manometric studies among the two studied groups

	Mean (SD)		Mean Difference (95 % CI)	t-statistics (df)^*^	*p*-value
	Control (*N* = 15)	Oral Tualang Honey (*N* = 15)			
Tensile Strength	187.33 (16.24)	241.33 (17.27)	-54.00 (-66.54, -41.56)	-8.82 (28)	<0.001
Histology Test: Histopathological grading among the two groups at day 7 after colonic anastomosis according to Ehrlich-Hunt numerical scale [22].					
Fibroblast count	1.93 (0.46)	2.87 (0.35)	-0.93 (-1.24, -0.63)	-6.26 (28)	<0.001
Inflammatory cells	2.87 (0.52)	2.27 (0.46)	-0.60 (-0.97, -0.24)	-3.37 (28)	0.002
Neovascularization	1.73 (0.59)	1.8 (0.86)	-0.07 (-0.62, 0.49)	-0.25 (28)	0.807

* Independent t test

## Results

**Fig. 1:**
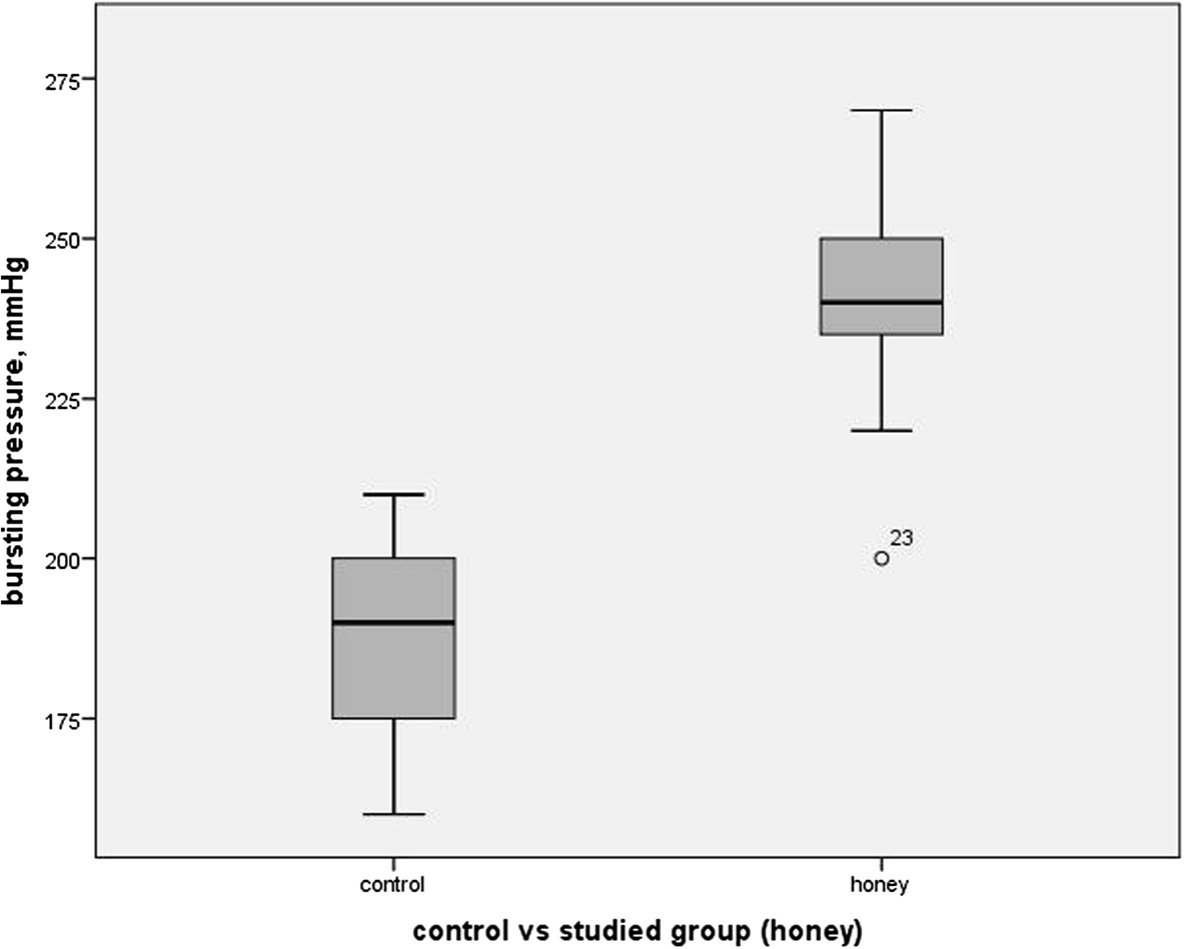
Comparison of bursting pressure (tensile strength) between groups treated with TH and control group

The results for the tensile strength by bursting pressure measurement between Group I (control) and Group II (study) are shown in Fig. 1. On the seventh day post-operatively, mean bursting pressure for Group II was significantly higher which was 241.33 mmHg (SD of 17.27) compared to Group I 187.33 mmHg (SD 16.24) with mean difference of -54.00 (CI 95 % -66.54,-41.46) (*p* < 0.001). There was a significantly higher fibroblast count in Group II with mean of 2.87 cells/mm^2^ (SD 0.35) compared to Group I with the mean of 1.93 cells/mm^2^ (SD 0.4) on the seventh day postoperatively. The difference between fibroblast count in both the groups was statistically significant (*p* < 0.001, 95 % CI -1.24,-0.63). Subjects treated with TH in the study group had statistically significant lower mean inflammatory cells count compared to those in control group with mean difference of -0.60 (95 % CI -0.60, -0.24, *p* = 0.002). There was no significant difference in neovascularization between both the groups. (95 % CI -0.62, 0.49, *p* = 0.807).

## Discussion

Honey is a supersaturated sugar solution, some of the components of which are added by the bees while the rest depend upon the plant origin from which the nectar has been collected, geographical location and the season. Acidity, hydrogen peroxide content, osmotic effect, nutritional and antioxidants content, stimulation of immunity, and unidentified compounds of honey are the factors responsible for its antibacterial activity; furthermore, by decreasing prostaglandin levels, elevating nitric oxide levels and exerting prebiotic effects the inflammatory and healing processes are also promoted [23]. However, no definite mechanism has been identified so far to explain the anti-inflammatory action of honey, which suggests that honey may have a number of effects on the molecular mechanisms of wound healing [15].

Anastomotic leakage, even under best circumstances, in colorectal surgery is approximately 3 % [24]. The efficiency of the topical application of honey on wounds or burns has been reported already in number of studies, however some of them exhibited that systemic administration of honey mainly intraperitoneal yields better results than topical treatment [25]. Current study focused on the oral administration of honey and used the bursting strength apart from histopathology examination to measure the healing of colonic anastomosis which is in accordance with study conducted by Nelson and Anders et al. [26] who advocated the use of bursting wall tension as a measure of bursting strength, considering it more accurate measurement. This finding was similar with study done by Gollu A. et al. in 2008 where they found that the colonic bursting pressures of the honey group (average:188.68 ± 14.26) were significantly better than the control group (average:149.72 ± 15.48, *p* = 0.001) [27]. Most studies evaluated that the maximum strength gained was seen between fourth to seventh day postoperatively. Jiborn H et al. (1978) found that on the seventh postoperative day, bursting strength values in the two study groups reached almost the same range as that of un-operated controls [28]. This finding implies that the anastomosis, in fact, is as strong as the surrounding wall and can be accomplished as early as the seventh postoperative day. In the current study, right side of colon was chosen because of the presence of cecum as a reference point for determining the site of bowel transection and anastomosis.

**Fig. 2:**
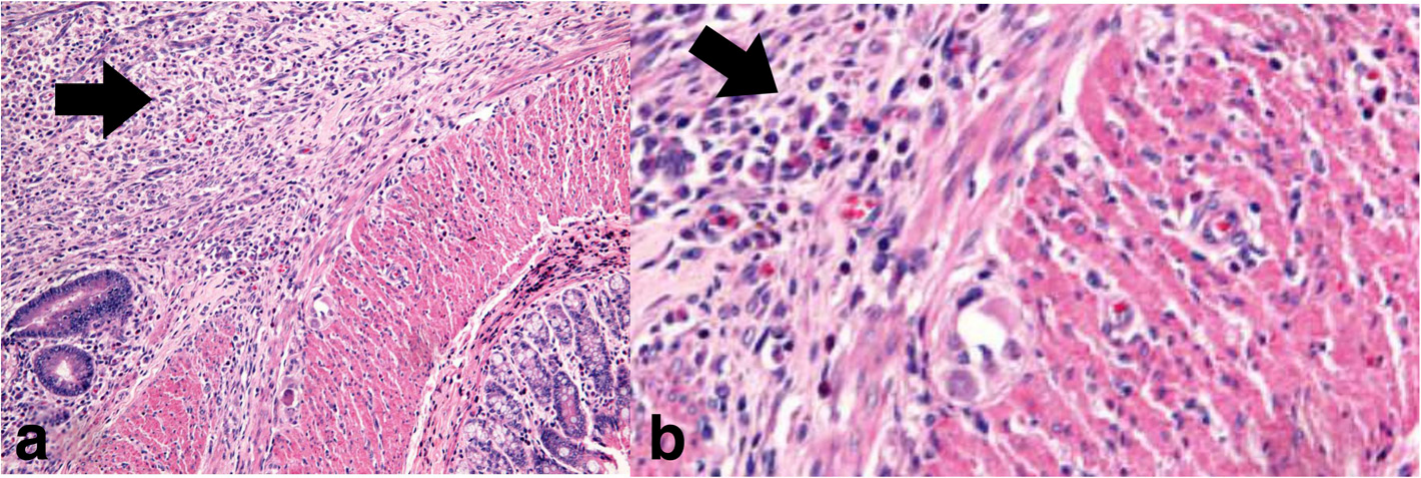
Lack of fibroblast count and abundance of inflammatory cells infiltration in control group [black arrows], H&E stain, X20(a), digitally zoomed 2× (b) (Courtesy of Pathology Department, HUSM)

**Fig. 3:**
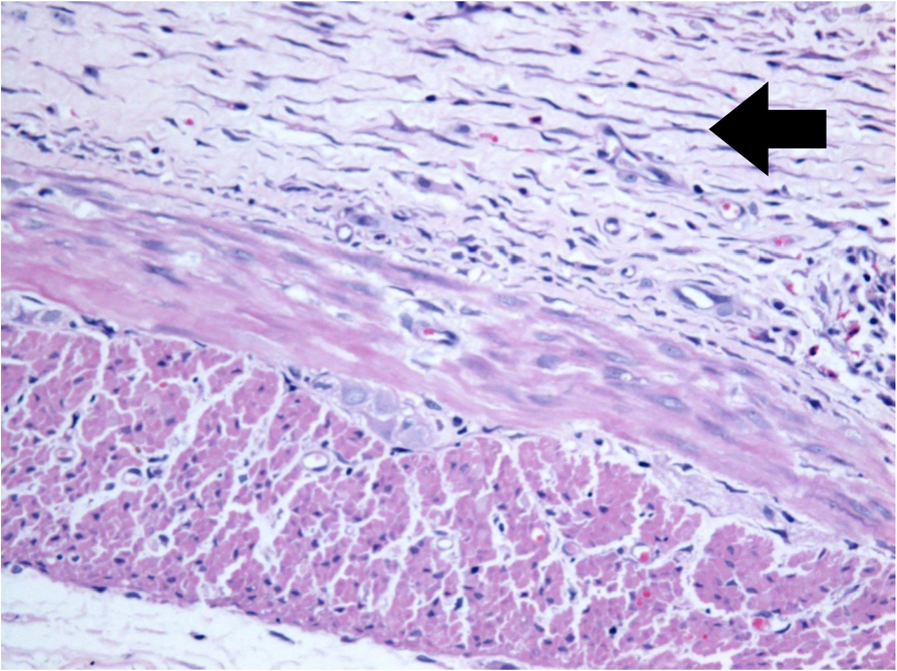
An increased fibroblast [black arrow] and less inflammatory cells infiltration in the TH group, H&E stain, X40 (Courtesy of Pathology Department, HUSM)

On the other hand, the recovery of tensile strength of healing skin is related to several factors including healing time and nutritional status of the healing tissues [29]. The increase in tensile strength of honey treated wounds could reflect not only the increased collagen content but also acceleration of collagen maturation resulting from the cross-linking of the collagen deposited by the fibroblasts. A slow increase in wound tensile strength corresponds to increase in fibroblasts, which begin to produce immature collagen during the proliferative phase of wound healing [30]. Because, fibroblasts play an important role in producing the collagen necessary to restore the tensile strength of wounded skin [31], the increased tensile strength in honey treated wound might be due to the increase in collagen concentration per unit area and stabilization of the fibrils [32]. Maturation of collagen fibrils resulted in stable cross links between several chains and these cross link are responsible for the gain in strength. In addition, honey was claimed to have high levels of glycine, methionine, arginine and proline which play definite roles in collagen formation and deposition [33]. Current study found that there was a significant higher fibroblasts count in TH group compared to the control groups on the day seven post treatment as seen in Fig. 2 and Fig. 3 which is in concordance with other studies which explained the similar phenomenon like in one study, in the propolis group, the proliferation, activation, and synthesis capacity of fibroblasts were much better than the control group and in another one, immature fibroblast were present on the first day post operatively in honey group and these cells matured and acquired the characteristic ultrastructural features of actively synthetic fibroblasts on the third day [34, 35].

Beside sugars, amino acids, minerals, vitamins and low level of hydrogen peroxide, honey, on the other hand, also contains some important components required to enhance synthesis of glycosaminoglycans which are one of the major components produced by fibroblasts in the wound area [15, 33]. Furthermore, TH possesses better antioxidant effects against a variety of reactive oxygen species when compared to other local Malaysian honeys like Gelam, Indian forest, and pineapple honeys [36].

Apparently in the current study, TH successfully showed statistically significant effect in lowering mean inflammatory cells count compared to those in control, however, it failed to statistically signify the slightly higher neovascularisation in those treated with TH compared to the control but it can be referred clinically significant as angiogenesis (which was actually high in TH group) eventually enhances wound healing process. This study failed to produce significant neovascularization as compared to another study conducted by Kilicoglu et al. [34], which may be attributed towards bigger sample size in that study compared to the current study. The dose of honey administered may be another factor involved regarding angiogenesis, as evident by multiple pilot studies, the best result on wound healing could be obtained at the dose of 1.5-2.0 ml orally plus 0.1-0.15 ml/cm^2^ topically or by topical application of 0.2-2.5 ml/cm^2^ [37]. However, overdosage caused an overgrowth and dehydration of the granulation tissue whereas lower doses resulted in less or no response; current study used 1.0 g/kg of Oral TH per day for 7 days postoperatively, which may also explain less angiogenesis as compared to other studies.

### Limitations

A little than usual dosage of TH was used so as to prevent overgrowth of the granulation tissue and also because further studies are still needed to come up with the optimum dose regimen regarding enteral feeding. There were some limitations of our study regarding cost which included the failure to assess collagen matrix production in the histopathology examination, only cell proliferation phase on day seven was assessed and also failure to attempt other types of biomechanical testing (e.g. elastic modulus).

### Further Research

Since the aim of this study was to evaluate the effects of TH in the enhancement of anastomotic healing, and not the mechanism of this effect, only possible mechanisms that might be responsible for positive effects of honey was hypothesized. Further studies are needed for the evaluation of the exact mechanism of healing. The bioavailability of honey should also be studied to evaluate the best dose for a better wound healing through enteral feeding and study regarding pre-operative administration of TH may also lead to a better understanding of the optimum treatment regimen. This experimental study included normal test subjects, further studies may look at malnourished or subjects with fecal contamination or peritonitis to evaluate the extent of effectiveness of TH in promoting wound healing. Results from the studies investigating the mechanism of action and controlled clinical trials, TH may be used for the prevention of anastomotic failure after major operations.

## Conclusion

Malaysian TH enhances not only the early phase of colon anastomotic healing by inhibiting the inflammatory response, but also stimulates the collagen synthesis of fibroblasts. The exact mechanisms of improved anastomotic wound healing by honey administration are not known, although increase of neoangiogenesis, mononuclear cell infiltration, and the stimulation of collagen synthesis are all involved.
